# Correction: Li et al. Identification and Functional Analysis of the Cell Proliferation Regulator, Insulin-like Growth Factor 1 (IGF1) in Freshwater Pearl Mussel (*Hyriopsis cumingii*). *Biology* 2022, *11*, 1369

**DOI:** 10.3390/biology12040548

**Published:** 2023-04-04

**Authors:** Xuenan Li, Shangle Feng, He Wang, Xiaoya Shen, Yige Chen, Xingrong Xuan, Yuanshuai Fu, Zhiyi Bai, Wenjuan Li

**Affiliations:** 1 Key Laboratory of Freshwater Aquatic Genetic Resources, Ministry of Agriculture and Rural Affairs, Shanghai Ocean University, Shanghai 201306, China; 2 Shanghai Collaborative Innovation for Aquatic Animal Genetics and Breeding, Shanghai 201306, China; 3 Shanghai Engineering Research Center of Aquaculture, Shanghai Ocean University, Shanghai 201306, China

## Figure Correction

In the original publication [1], the original versions of Figures 3, 4 and 7 were mistakenly published as the final versions. Below are correct versions of Figure 3, Figure 4 and Figure 7. The authors state that the scientific conclusions are unaffected. The original publication has also been updated.

## Figures and Tables

**Figure 3:**
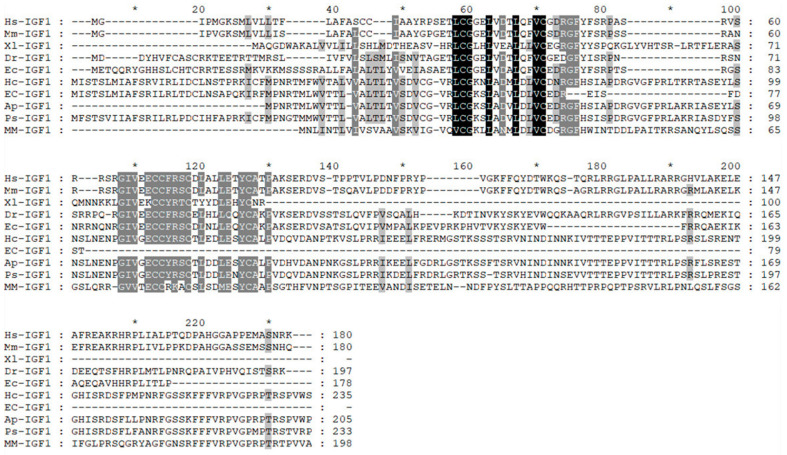
Sequence comparison of homology of IGF1 with other species with *H. cumingii.* Other species NCBI accession numbers: *Homo sapiens* (Hs): NP_000609.1; *Mus musculus* (Mm): NP_001300939.1; *Xenopus laevis* (Xl): NP_001156865.1; *Danio rerio* (Dr): NP_571900.1; *Epinephelus coioides* (Ec): AMR58932.1. *Elliptio complanate* (EC): GAHW01021065.1; *Amblema plicata* (Ap): GITL01141792.1; *Potamilus streckersoni* (Ps): GJAA01011606.1; *Mercenaria mercenaria* (MM): XP_045198864.1. Black: conserved amino acid residue; gray: analogous residues.

**Figure 4:**
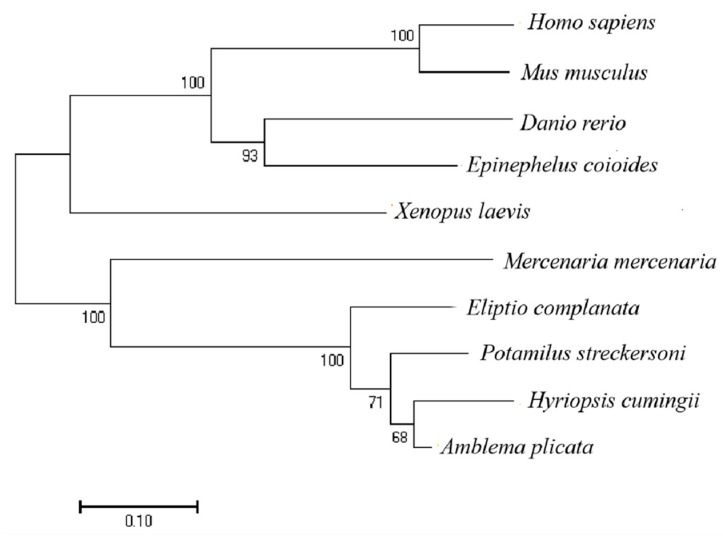
Phylogenetic analysis of IGF1 protein from different species. GenBank accession numbers are as in Figure 3, the number on the node indicates the confidence value of the test for 1000 bootstrap repetitions.

**Figure 7:**
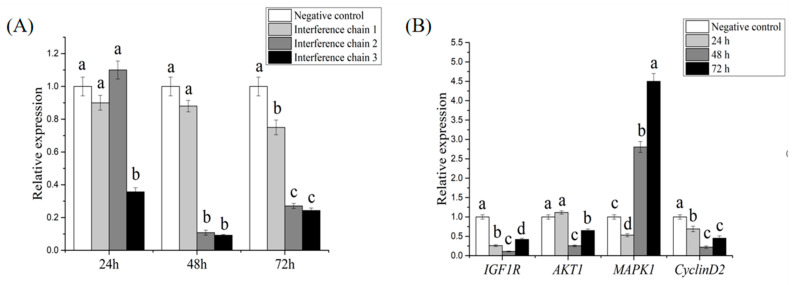
(**A**) Relative expression levels of *HcIGF1* in the mantle after interference. Interference chains 1, 2, and 3 represent G1, G2, and G3, respectively. Different letters at the same time point indicate significant differences (*p* < 0.05). (**B**) Relative expression of downstream genes of *HcIGF1* in the mantle after interference. Different letters for the same gene indicate significant differences (*p* < 0.05).
