# Supplementary figures and images for: Identification and Functional Analysis of the Cell Proliferation Regulator, Insulin-like Growth Factor 1 (IGF1) in Freshwater Pearl Mussel (Hyriopsis cumingii)
Source: Biology (Basel). 2022 Sep 19;11(9):1369. doi: 10.3390/biology11091369 (PMC9495379; doi:10.3390/biology11091369)

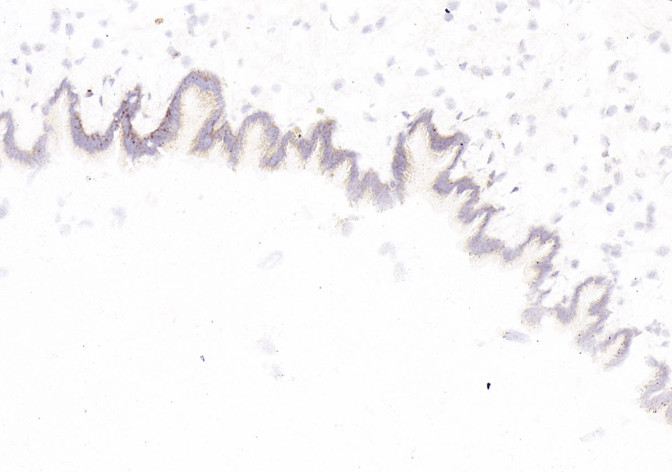

Supplement: Supplementary file 1 [file biology-11-01369-s001.zip › Figure S1-Figure 6 ú¿10xú⌐.jpg]
